# Nicotinamide mononucleotide protects STAT1 from oxidative stress‐induced degradation to prevent colorectal tumorigenesis

**DOI:** 10.1002/mco2.70006

**Published:** 2024-11-21

**Authors:** Ting Li, Chengting Luo, Zongyuan Liu, Jinyu Li, Meng Han, Ran Zhang, Yuling Chen, Haiteng Deng

**Affiliations:** ^1^ MOE Key Laboratory of Bioinformatics, Center for Synthetic and Systematic Biology, School of Life Sciences Tsinghua University Beijing China; ^2^ School of Life Science and Technology Wuhan Polytechnic University Wuhan China; ^3^ School of Life Science Yunnan University Yunnan China

**Keywords:** colorectal cancer, cysteine trioxidation, nicotinamide mononucleotide, STAT1

## Abstract

Colitis, accompanied by the accumulation of reactive oxygen species (ROS) in the intestinal tract, is a risk factor for colorectal cancer (CRC). Our previous studies indicate that nicotinamide mononucleotide (NMN) replenishment reduces chronic inflammation. In this study, we confirm that NMN supplementation reduces inflammatory cytokine levels and oxidative tissue damage in an azoxymethane/dextran sulfate sodium (AOM/DSS)‐induced colitis‐associated cancer (CAC) model. Mice treated with NMN developed fewer colon tumors than untreated animals under the same AOM/DSS treatment conditions. Quantitative proteomic analysis revealed a decrease in signal transducer and activator of transcription 1 (STAT1) expression in the CAC model. We demonstrate that STAT1 overexpression induces G1 arrest by downregulating CDK6 expression and suppressing tumor cell proliferation and migration. Of note, H_2_O_2_ induced trioxidation of the STAT1 protein and promoted its degradation, which was partially reversed by NMN supplementation. Upon H_2_O_2_ treatment, Cys155 in STAT1 was oxidized to sulfonic acid, whereas the mutation of Cys155 to alanine abolished ROS‐mediated STAT1 degradation. These results indicate that oxidative stress induces STAT1 degradation in tumor cells and possibly in CAC tissues, whereas supplementation with NMN protects STAT1 from oxidation‐induced degradation and prevents tumorigenesis. This study provides experimental evidence for the development of NMN‐mediated chemoprevention strategies for CRC.

## Graphical abstract

Nicotinamide mononucleotide (NMN) supplementation reduces tumorigenesis in a mouse azoxymethane/dextran sulfate sodium (AOM/DSS)‐induced colorectal cancer (CRC) model. Signal transducer and activator of transcription 1 (STAT1) protein levels are decreased in AOM/DSS‐treated mice, with higher levels maintained upon NMN supplementation. Mechanistically, tumorigenesis‐associated reactive oxygen species (ROS) induce cysteine residue peroxidation in STAT1, inducing its degradation. NMN supplementation reduces STAT1 degradation by decreasing intracellular ROS levels. Following nuclear translocation, STAT1 regulates downstream gene expression, inhibiting CRC cell proliferation and migration.

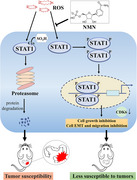

## 1 INTRODUCTION

Colorectal cancer (CRC) is the third leading cause of cancer‐related deaths worldwide.^1^ Colitis‐associated cancer (CAC), a particularly aggressive subtype of CRC, develops in patients with inflammatory bowel disease owing to long‐term exposure to chronic inflammation.^2^ During inflammation, activated immune cells are recruited to the site of injury, where increased cellular respiration leads to increased release and accumulation of reactive oxygen species (ROS).^3^ Sustained oxidative stress increases the oxidative modification of nucleic acids, lipids, and proteins, which increases the risk of mutagenesis and leads to tumorigenesis.^4^ CRC comprises multistep, multipathway progression from early adenoma to cancer and usually takes 10–15 years to advance, thus providing an opportunity for early detection and prevention; moreover, its high morbidity and mortality rates highlight the importance of prevention.^5^ In the 1970s, chemoprevention was recognized as a potential strategy to reduce the incidence of cancer and cancer‐related deaths. Chemopreventive drugs have become a core focus in cancer prevention research given their safety, efficacy, and ease of use. Additionally, various natural and synthetic molecules have been investigated as potential CRC chemopreventive agents, including aspirin, nonsteroidal anti‐inflammatory drugs, 5‐aminosalicylic acids, ursodeoxycholic acid, metformin, statins, long‐chain omega‐3 polyunsaturated fatty acids, folic acid, and antioxidants such as selenium, vitamins A, C, and E, β‐carotene, and curcumin.^6^ Although some chemopreventive agents have shown potential to reduce the risk of adenomas and CRC in clinical trials, the results have been inconsistent, and some drugs have limited use as chemopreventive agents owing to side effects. Therefore, further research is needed to identify ideal chemopreventive agents, as well as their dosage, mode of administration, and potential biomarkers.

Nicotinamide mononucleotide (NMN) is an immediate precursor of nicotinamide adenine dinucleotide (NAD^+^) synthesis. Impaired DNA damage repair and declining immune function due to decreased NAD^+^ levels are important triggers of cancer, and slowing the aging of an organism can prevent tumors to a certain extent. Research indicates that NAD^+^ precursors could be used to treat tumors that are unresponsive to immunotherapy by increasing NAD^+^ levels. NAD^+^ metabolism can trigger cancer immune evasion by enhancing PD‐L1 on cancer cells; combining NMN with a PD‐L1 antibody notably slows tumor growth in living organisms.^7^ Additionally, NMN treatment increases natural killer (NK) cell activity and significantly inhibits melanoma growth.^8^ However, whether NMN supplementation protects against CRC development has not been tested. Here, we report that NMN supplementation prevents the development of azoxymethane/dextran sulfate sodium (AOM/DSS)‐induced CAC in mice. Mechanistically, we found that the protective effect of NMN supplementation was mediated, at least in part, by the maintenance of signal transducer and activator of transcription 1 (STAT1) levels and a reduction in inflammation under oxidative stress.

Signal transducer and activator of transcription family members can regulate the expression of genes involved in cell growth, differentiation, and apoptosis. A large body of evidence shows aberrant expression of STAT1 in a variety of mammalian malignancies, including ovarian cancer,^9^ pancreatic cancer,^10^ and CRC.^11^ STAT1 inhibits CRC progression by counteracting the tumorigenic STAT3 signaling pathway.^11^ STAT1 deficiency promoted CRC development in mice.^12^ Furthermore, CRC patients with higher colon STAT1 expression had a better prognosis than those with lower STAT1 expression.^13^ Therefore, maintaining STAT1 activity is a promising therapeutic strategy for CRC. However, how STAT1 protein expression is downregulated during CRC progression remains poorly understood.

Many amino acid residues in proteins, especially sulfur‐containing amino acids, such as cysteine (Cys) and methionine, are highly susceptible to oxidation by ROS in living organisms. Under sustained oxidative stress, ROS oxidize the sulfhydryl groups of amino acids to subsulfonic (–SOH), sulfinic (–SO_2_H), and sulfonic (–SO_3_H) acids.^14^ These different protein oxidation states can lead to different biological structures, and changes in the protein conformation, as well as the formation of protein polymers and aggregates, can lead to significant changes in solubility and functionality, thus altering cell fates. Our previous study showed that the cystine trioxidation of 15‐hydroxyprostaglandin dehydrogenase (15‐PGDH) at Cys44 leads to its degradation.^15^ STAT1, a protein sensitive to redox changes, exhibits cell‐specific characteristics.^16^ For example, H_2_O_2_ treatment inhibits STAT1 phosphorylation in neuronal cells but not in non‐neuronal cells.^17^ In this study, we confirmed that ROS induces STAT1 degradation in tumor cells, suggesting that STAT1 degradation during CAC progression might be induced by ROS. Crucially, NMN supplementation restored STAT1 protein levels under oxidative stress. Thus, targeting STAT1 via NMN supplementation could be an effective strategy for preventing CRC.

## 2 RESULTS

### 2.1 NMN attenuates AOM/DSS‐induced colon tumorigenesis in mice

**FIGURE 1:**
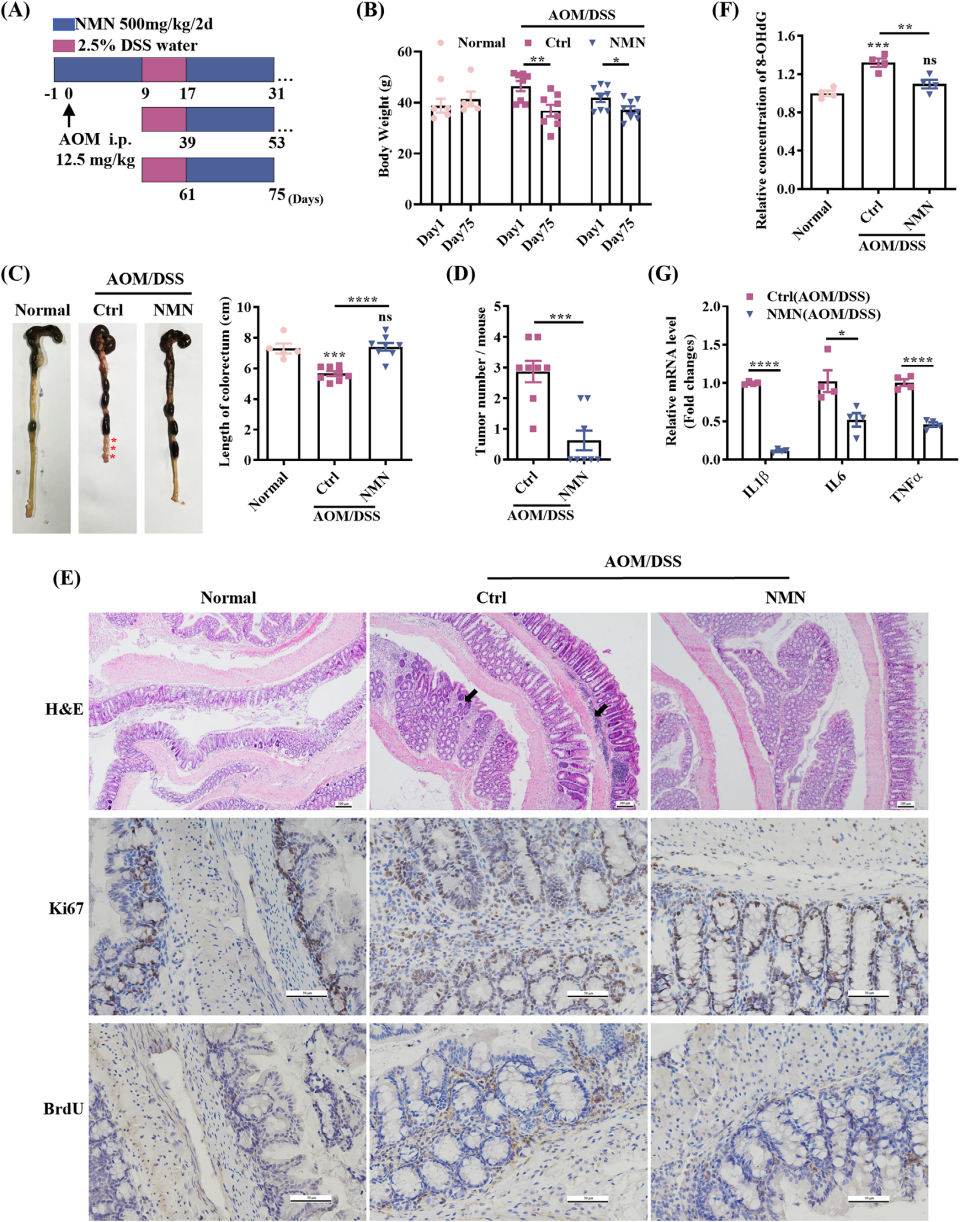
Nicotinamide mononucleotide (NMN) supplementation reduces the colon tumorigenesis in azoxymethane/dextran sulfate sodium (AOM/DSS) mouse model of colorectal cancer (CRC). (A) Schematic timeline for AOM/DSS‐induced mouse model. (B) AOM/DSS induced a significant decrease in mice body weight. (C) Mark the location of the tumor nodules with a red asterisk. The colon length was significantly shortened in the AOM/DSS control group. (D) Colon tumors in control and NMN‐treated mice after AOM injection. More tumors were observed in the AOM/DSS control group than in the NMN‐treated group. (E) Histopathological analysis by hematoxylin and eosin staining. Scale bar: 100 µm. Proliferation state was indicated by immunohistochemistry staining for Ki67 and BrdU. Scale bar: 50 µm. AOM/DSS‐induced destruction of intestinal epithelial cells, damage of the intestinal barrier integrity, and extensive infiltration of inflammatory cells (black arrows), which were significantly relieved by NMN treatment. (F) The relative content of 8‐hydroxy‐2′‐deoxyguanosine (8‐OHdG) in mouse colorectal tissues. (G) mRNA expression levels of inflammatory factors in mice's colon tissues were measured by quantitative polymerase chain reaction (qPCR). Means ± SEM, *p < 0.05, **p < 0.01, ***p < 0.001, ****p < 0.0001; ns, not significant; Student's *t*‐test or one‐way analysis of variance (ANOVA) test. H&E, hematoxylin and eosin.

To determine whether NMN attenuates tumorigenesis in mice with CAC, we first examined its effects in an established mouse model. In this model, mice were intraperitoneally injected with the procarcinogen AOM, followed by three rounds of DSS administration to induce intestinal inflammation and tumorigenesis in the distal colon.^18^ In the AOM/DSS NMN‐treated group, NMN treatment was initiated the day before AOM administration, and NMN injections were performed every other day until the end of the experiment (Figure 1A). After drinking water containing 2.5% DSS for several days, the mice showed a significantly increased number of feces and diarrhea symptoms, whereas no diarrhea was observed in the NMN‐treated group (Figure S1A). Significant weight loss was also observed in the AOM/DSS control group, whereas the weight loss trend was more moderate in the AOM/DSS NMN‐treated group (Figures 1B and S1B). This shows that AOM/DSS treatment impairs the intestinal barrier, while NMN supplementation helps safeguard intestinal function against the detrimental effects of carcinogens and inflammatory agents. Additionally, NMN treatment prevented AOM/DSS‐induced colon shortening (Figure 1C) and tumor nodule formation (Figure 1D). Figure S1C shows that the distal colon of mice in the AOM/DSS control group exhibited swelling and hemorrhage. Conversely, the intestinal walls of the mice in the AOM/DSS NMN‐treated group were more intact and resilient, with no significant damage. Histological analysis showed that NMN treatment alleviated epithelial hyperplasia, as evidenced by a reduction in immunohistochemical staining for Ki67 and BrdU (Figures 1E and S1D,E). Intact mucosal epithelial cells and normal structural morphology of the muscularis were observed in the normal group. Increased inflammatory cell infiltration and glandular damage were observed in the AOM/DSS control group (Figure 1E). To determine the extent of oxidative damage to the intestinal tract induced by AOM/DSS, we measured the levels of 8‐hydroxy‐2′‐deoxyguanosine (8‐OHdG) generated after the repair of ROS‐mediated DNA damage. The results showed that NMN supplementation significantly reduced AOM/DSS‐induced oxidative damage, whereas 8‐OHdG levels were higher in CAC tissues (Figure 1F). Since inflammation plays a critical role in tumorigenesis,^19^ we examined the expression levels of proinflammatory cytokines in mouse colon tissues using quantitative polymerase chain reaction (qPCR). The results show that NMN reduced the expression levels of interleukin (IL)‐1β, IL‐6, and tumor necrosis factor (TNF)‐α in mouse colon after AOM/DSS treatment (Figure 1G).

### 2.2 NMN restores STAT1 protein levels in CAC tissues

**FIGURE 2:**
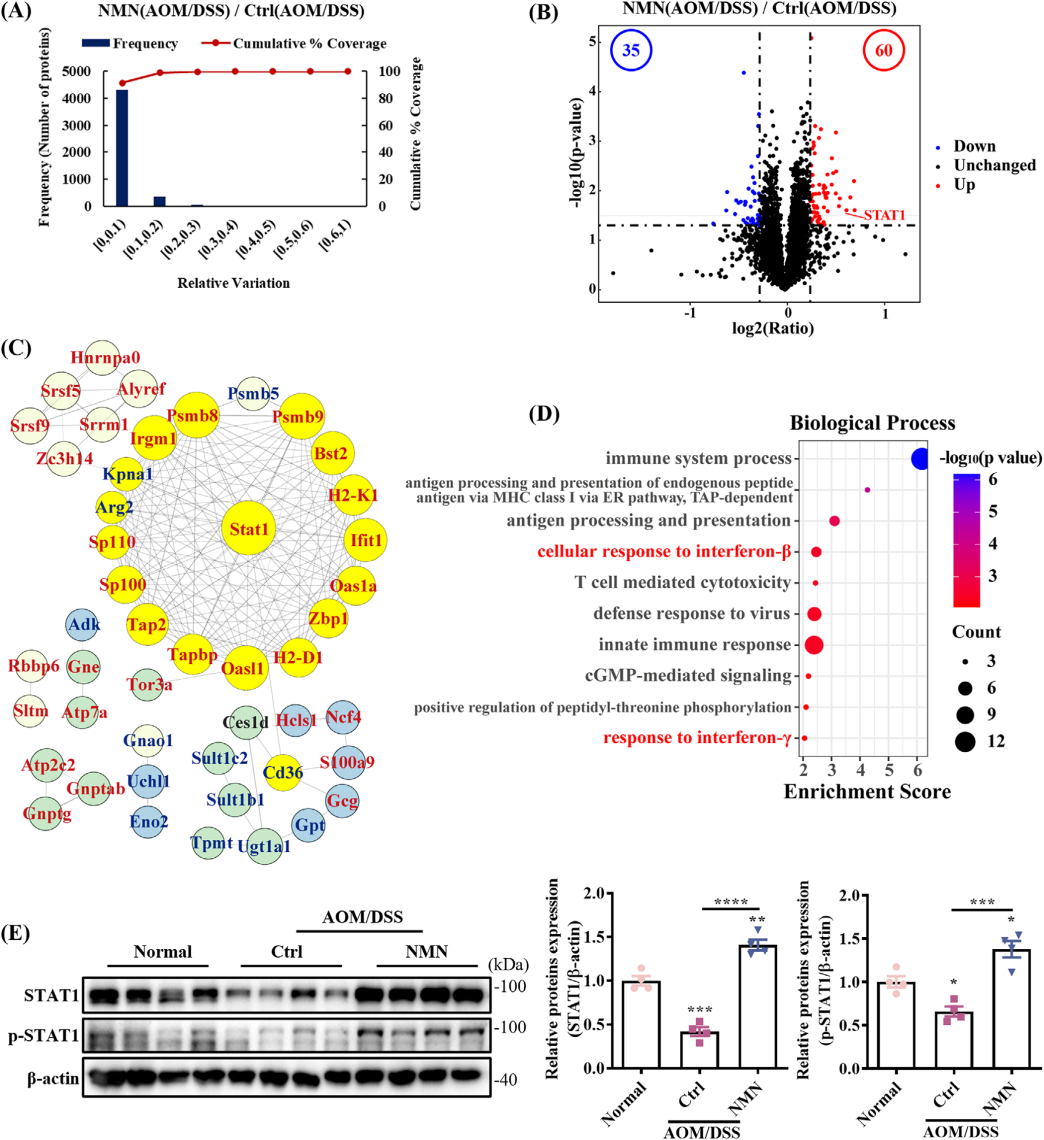
Nicotinamide mononucleotide (NMN) treatment restores signal transducer and activator of transcription 1 (STAT1) level in the colitis‐associated cancer (CAC) tissues. (A) Experimental variations of colon proteomics analysis between the control and NMN‐treated groups under azoxymethane/dextran sulfate sodium (AOM/DSS) condition. (B) Volcano plot representing differences in proteins between the control and NMN‐treated groups. (C) Protein–protein interactions of differentially expressed proteins (DEPs) constructed using Cytoscape (V3.10.0). Red and blue fonts indicate upregulated and downregulated proteins, respectively. Yellow nodes are proteins that interact with STAT1. (D) Gene ontology (GO) annotation of DEPs. (E) Western blotting verification of STAT1 and phosphorylated STAT1. Means ± SEM, *p < 0.05, **p < 0.01, ***p < 0.001, ****p < 0.0001; one‐way analysis of variance (ANOVA) test.

To investigate the mechanism by which NMN treatment reduces colon tumorigenesis, we performed quantitative proteomic analysis to characterize proteomic changes in the colon. We assessed 6814 proteins, of which 4706 were confidently identified (false discovery rate [FDR] of protein <0.01, sum posterior error probability [PEP] score ≥5, unique peptide ≥2) by Protein Discovery 2.3 (Table S1). Principal component analysis (PCA) demonstrated clear separation between groups, with greater intergroup than intragroup variation (Figure S2A). According to a previous study,^20^ the percentage variation corresponding to 88% coverage was considered the threshold cutoff (Figure 2A). Thus, proteins with a ratio of ≥1.2 or ≤0.83 (*p* value <0.05) were considered upregulated or downregulated proteins, respectively. Among the differentially expressed proteins (DEPs), 60 were upregulated and 35 were downregulated in the AOM/DSS NMN‐treated group compared to the AOM/DSS control group (Figure 2B and Table S2). Based on these DEPs, STRING analysis revealed enrichment of proteins involved in antigen processing and presentation, with transporter associated with antigen processing (TAP) complex‐binding proteins and proteasome subunits identified as central in the interaction network by Cytoscape (Figure 2C). Most of these proteins interacted with STAT1 (Figure 2C). Gene ontology (GO) annotation indicated that these proteins were mainly major histocompatibility complex (MHC) class I peptide‐loading complexes, which are essential for the immune response (Figure S2B,D).

Biological processes involved cellular responses to interferon (IFN) signaling pathways, particularly IFN‐β and IFN‐γ, with STAT1 as the key effector (Figure 2D). As shown in Figure S2C, the expression of several proteins involved in IFN signaling pathway was upregulated. Therefore, we investigated STAT1 expression in the mouse colon. The protein levels of STAT1 in the normal and AOM/DSS NMN‐treated groups were significantly higher than those in the AOM/DSS control group (Figure 2E). Under signaling stimulation, STAT1 typically undergoes phosphorylation and is subsequently translocated to the nucleus to promote the transcription of target genes. Therefore, we examined the phosphorylation of STAT1. The results showed that the phosphorylation level of STAT1 was decreased in CAC mice, whereas supplementation with NMN maintained the phosphorylation level of STAT1 (Figure 2E). We also examined STAT3 and its phosphorylation levels, but the results showed that neither was significantly altered in the CAC mouse model (Figure S2E). However, there were no clear differences in the mRNA levels of STAT1 and STAT3 among the three groups (Figure S2F,G). These results indicate that NMN treatment inhibited the degradation of STAT1 in the CAC model.

### 2.3 ROS‐induced degradation of STAT1

**FIGURE 3:**
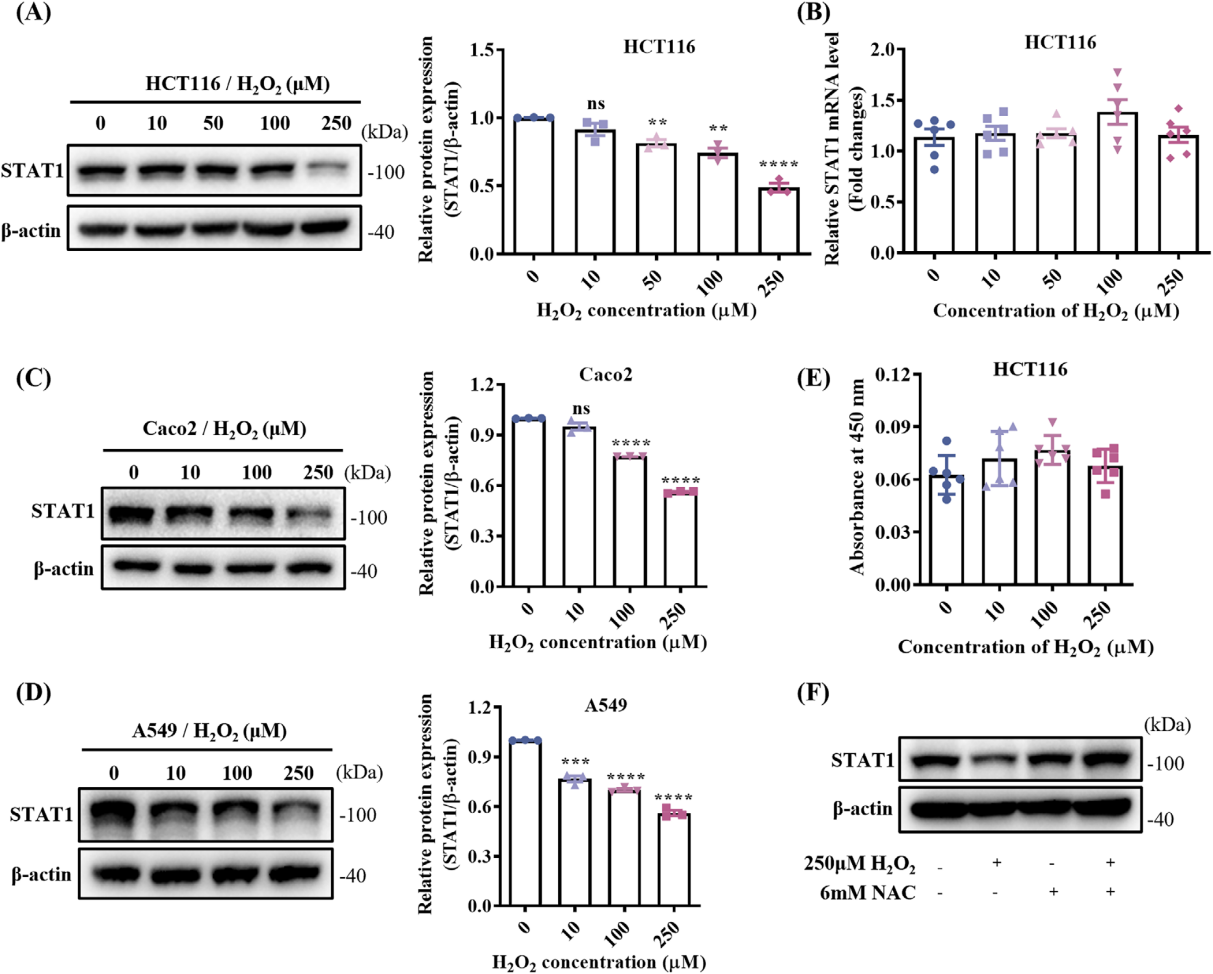
Reactive oxygen species (ROS)‐induced degradation of signal transducer and activator of transcription 1 (STAT1). The expression levels of STAT1 in HCT116 (A), Caco2 (C), and A549 (D) cells treated with different concentrations of H_2_O_2_ were detected by Western blot (WB), respectively. (B) Quantitative polymerase chain reaction (qPCR) results showed that the STAT1 mRNA levels did not change under H_2_O_2_ treatment in HCT116 cells. (E) The proliferation of HCT116 cells was not affected by different concentrations of H_2_O_2_ treatment for 12 h. (F) The expression of STAT1 in untreated and H_2_O_2_‐treated HCT116 cells with or without pretreatment with 6 mM N‐acetylcysteine (NAC). Means ± SEM, **p < 0.01, ***p < 0.001, ****p < 0.0001; ns, not significant; one‐way analysis of variance (ANOVA) test.

To investigate the reason for the decreased STAT1 protein level in the CAC mouse model, HCT116 cells were treated with different concentrations of H_2_O_2_, and the results showed that the protein level of STAT1 decreased with the concentration of H_2_O_2_ after 12 h of treatment, which decreased to half that of the untreated group at the concentration of 250 µM (Figure 3A). In contrast, the qPCR results showed that H_2_O_2_ had no effect on STAT1 mRNA levels (Figure 3B). To determine if this phenomenon is common, we repeated the experiments in colorectal adenocarcinoma Caco2 and lung carcinoma A549 epithelial‐like cells. Consistent results were obtained in both cell lines, that is, a decreased protein level of STAT1 was induced by H_2_O_2_, but not the mRNA level in both Caco2 (Figures 3C and S3A) and A549 (Figures 3D and S3B) cells. In addition, short‐term H_2_O_2_ treatment (12 h) did not affect cell growth (Figure 3E). Pretreatment with 6 mM N‐acetylcysteine (NAC) for 6 h prior to H_2_O_2_ addition partially blocked ROS‐induced STAT1 degradation in HCT116 cells (Figures 3F and S3C), confirming ROS as the cause.

To determine the pathways behind ROS‐mediated STAT1 degradation, cells were pretreated with MG132, a proteasome inhibitor, which partially prevented STAT1 degradation in HCT116 cells after 6 h of treatment (Figure S3D). Next, we immunoprecipitated STAT1 complexes from H_2_O_2_‐treated and untreated HCT116‐STAT1‐OE cells to identify possible modifications. As expected, H_2_O_2_ treatment resulted in increased levels of STAT1 ubiquitination (Figure S3E). We also investigated the STAT1 interactome by immunoprecipitation of STAT1 following H_2_O_2_ treatment. Proteins with ratios greater than 1.3 or less than 0.76 (sum PEP score ≥5, unique peptides ≥2) were considered DEPs, which included 1689 upregulated proteins and 207 downregulated proteins (Table S3). Kyoto Encyclopedia of Genes and Genomes (KEGG) enrichment analysis of the DEPs included the proteasome pathway (Figure S3F), and STAT1 bound to multiple 26S proteasome regulatory subunits (Figure S3G), most of which were upregulated upon H_2_O_2_ stimulation. Furthermore, we found that STAT1 binds to the E3 ligase, TRIM21, which was recently reported to mediate the ubiquitination degradation of STAT1,^21^ suggesting that the ubiquitin–proteasome pathway is involved in the ROS‐induced degradation of STAT1.

### 2.4 NMN inhibits ROS‐induced degradation of STAT1

**FIGURE 4:**
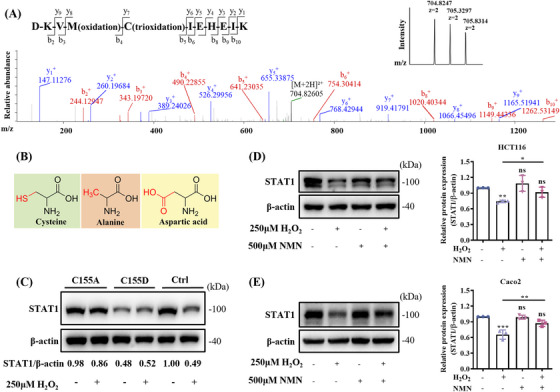
Nicotinamide mononucleotide (NMN) supplementation prevents the oxidative degradation of signal transducer and activator of transcription 1 (STAT1). (A) Tandem mass spectrometry spectrum of the sulfonated peptide showing the identification of Cys155 modifications in STAT1 in the presence of H_2_O_2_, with inset showing the intensity of the primary spectrum of the peptide. (B) The structures of cysteine (C), alanine (A), and aspartic acid (D). (C) Western blotting of H_2_O_2_‐treated and untreated HCT116 cells transfected with flag‐tagged STAT1, STAT1‐C155A, or STAT1‐C155D. Relative optical densities of STAT1 blots normalized to β‐actin are indicated below the blots. Five hundred µM NMN treatment prevented the reduction of STAT1 protein induced by H_2_O_2_ treatment in HCT116 (D) and Caco2 (E) cells. Means ± SEM, *p < 0.05, **p < 0.01, ***p < 0.001; ns, not significant; one‐way analysis of variance (ANOVA) test.

Notably, Cys155 in STAT1 was oxidized to sulfonic acid upon H_2_O_2_ treatment (Figure 4A). In addition, the oxidation of the Cys155 residue to sulfonic acid in STAT1 was increased under 250 µM H_2_O_2_ stimulation compared to 100 µM H_2_O_2_ (Figure S4A). Considering that cysteine trioxidation triggered protein degradation,^15^ site‐directed mutagenesis was used to verify Cys155's role in STAT1 degradation. Given the high reactivity of the sulfhydryl group, alanine (A) and aspartate (D) were selected to represent the reduced and oxidized states of cysteine, respectively (Figure 4B). HCT116 cells were transfected with DNA constructs harboring the wild‐type STAT1 gene or the Cys155A and Cys155D mutants, then treated with H_2_O_2_. Equal amounts of protein from both treated and untreated cells were analyzed by sodium dodecyl‐sulfate polyacrylamide gel electrophoresis (SDS‐PAGE) and Western blot (WB) analysis. The findings indicated that the Cys155A mutation halted ROS‐mediated STAT1 degradation (Figure 4C), confirming that Cys155 is critical for ROS‐induced STAT1 degradation. In contrast, the mutation of Cys155 to aspartate, mimicking oxidized cysteine, triggered STAT1 degradation, even without H_2_O_2_ treatment, and STAT1 expression levels did not further decrease upon H_2_O_2_ treatment (Figure 4C). These results suggest that oxidative modification of Cys155 plays a critical role in triggering STAT1 degradation.

Moreover, we showed that NMN treatment reduced the ROS levels in HCT116 cells (Figure S4B). When we treated HCT116 cells with both H_2_O_2_ and NMN, the ROS‐induced decrease in STAT1 expression was partially inhibited by NMN supplementation (Figure 4D). Moreover, a 12‐h NMN treatment showed no impact on cell proliferation (Figure S4C). The protective role of NMN against STAT1 degradation was similarly observed in Caco2 (Figure 4E) and A549 (Figure S4D) cells. These results suggest that NMN supplementation prevents STAT1 degradation induced by H_2_O_2_ treatment.

### 2.5 STAT1 overexpression inhibits CRC cell proliferation and migration

**FIGURE 5:**
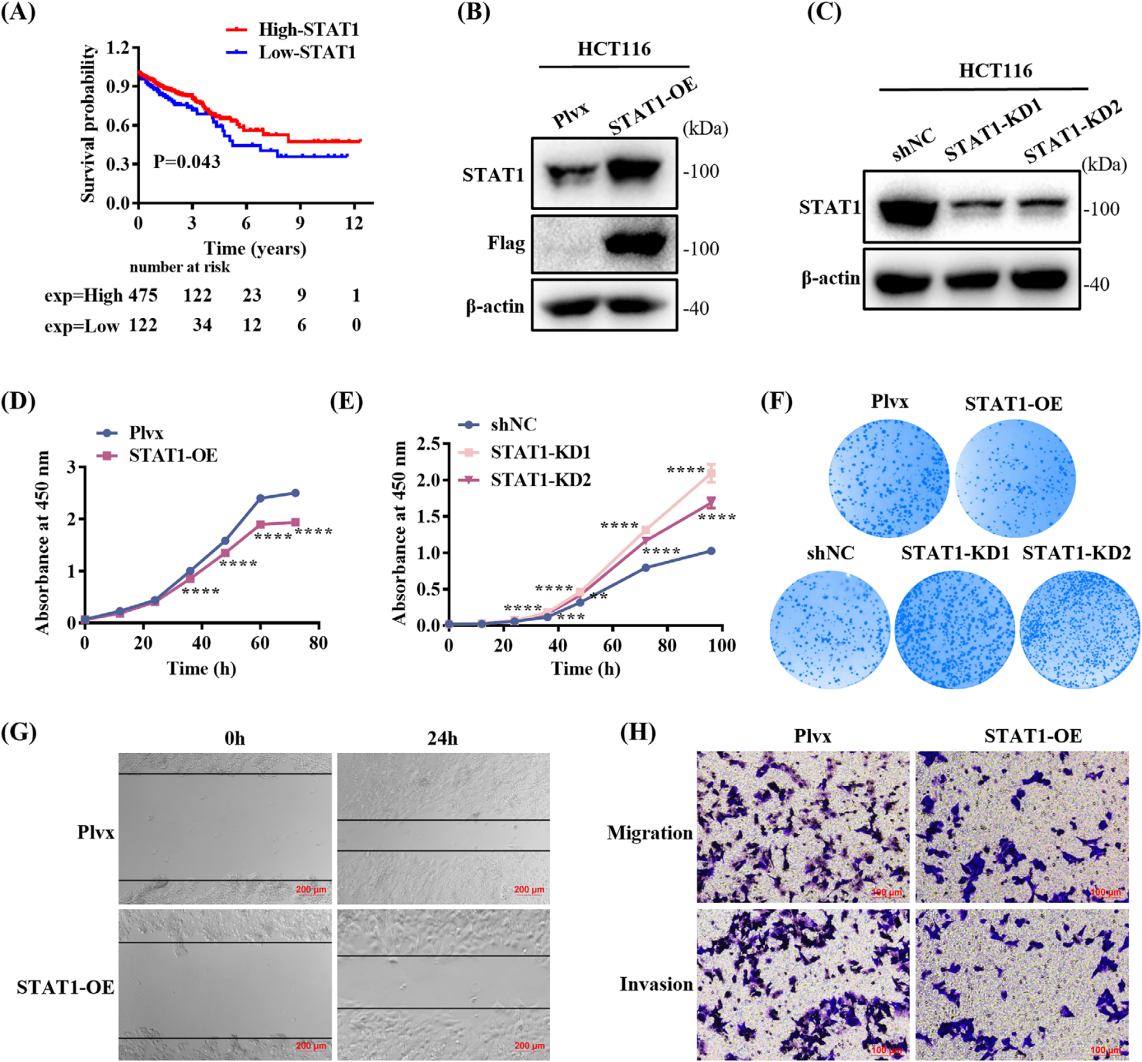
Signal transducer and activator of transcription 1 (STAT1) overexpression inhibits cell proliferation and migration. (A) Kaplan–Meier overall survival analysis between patients with high (*n* = 475) and low (*n* = 122) STAT1 expression. *p* value was calculated using log‐rank test. (B) STAT1 overexpression verification by Western blot (WB). (C) STAT1 knockdown verification by WB. (D) STAT1 overexpression in HCT116 cells inhibited cell growth. (E) STAT1 knockdown promoted cell proliferation. (F) STAT1 overexpression impairs colony formation ability in HCT116, whereas STAT1 knockdown promotes colony formation. (G) Cell migration was inhibited in HCT116‐STAT1‐OE cells compared with that in the control cells. Scale bar: 200 µM. (H) Transwell assays of cell migration and invasion in STAT1‐overexpressing cells. Scale bar: 100 µM. Means ± SEM, **p < 0.01, ***p < 0.001, ****p < 0.0001; Student's *t*‐test or one‐way analysis of variance (ANOVA) test.

To investigate the relationship between STAT1 expression and prognosis in CRC patients, we analyzed the transcriptome data of 597 CRC patients from The Cancer Genome Atlas (TCGA; Figure 5A). The results showed that high STAT1 expression was positively associated with prolonged survival. To confirm the inhibitory effect of STAT1 on CRC cells, we constructed a cell line (HCT116‐STAT1‐OE) in which STAT1 was stably overexpressed by lentiviral infection; cells transduced with the lentiviral vector encoding the empty pLVX‐IRES‐ZsGreen1 cassette were used as the control (HCT116‐Plvx). Furthermore, two different short hairpin RNAs (shRNAs) against STAT1 were used to establish stable STAT1 knockdown cell lines, designated HCT116‐STAT1‐KD1 and HCT116‐STAT1‐KD2. Cells transfected with an empty vector plasmid were used as negative controls (HCT116‐shNC). qPCR and WB results showed that STAT1 overexpression was significantly higher in HCT116‐STAT1‐OE cells than that in control cells (Figures S5A and 5B), and STAT1 expression was reduced (Figures S5A and 5C) in knockdown cells. CCK‐8 assays showed that STAT1 overexpression reduced the cell growth rate (Figure 5D), whereas STAT1 knockdown promoted cell growth (Figure 5E). The colony formation assay showed that the HCT116‐STAT1‐OE group had fewer colonies than the control group, whereas STAT1 knockdown promoted colony formation (Figures 5F and S5B). In addition, the migration distance of STAT1‐overexpressing cells was significantly shortened over the same period as that of control cells (Figures 5G and S5C), while the result was reversed in STAT1 knockdown cells (Figure S5D,E). We also performed Transwell assays to demonstrate the effects of STAT1 on the migration and invasion of CRC cells (Figures 5H and S5F). The results show that the number of STAT1‐overexpressing cells passing through the chamber was relatively low, whereas STAT1 knockdown promoted the migration and invasion of HCT116 cells (Figure S5G,H).

### 2.6 STAT1 decreases CDK6 expression and inhibits epithelial–mesenchymal transformation in CRC cells

**FIGURE 6:**
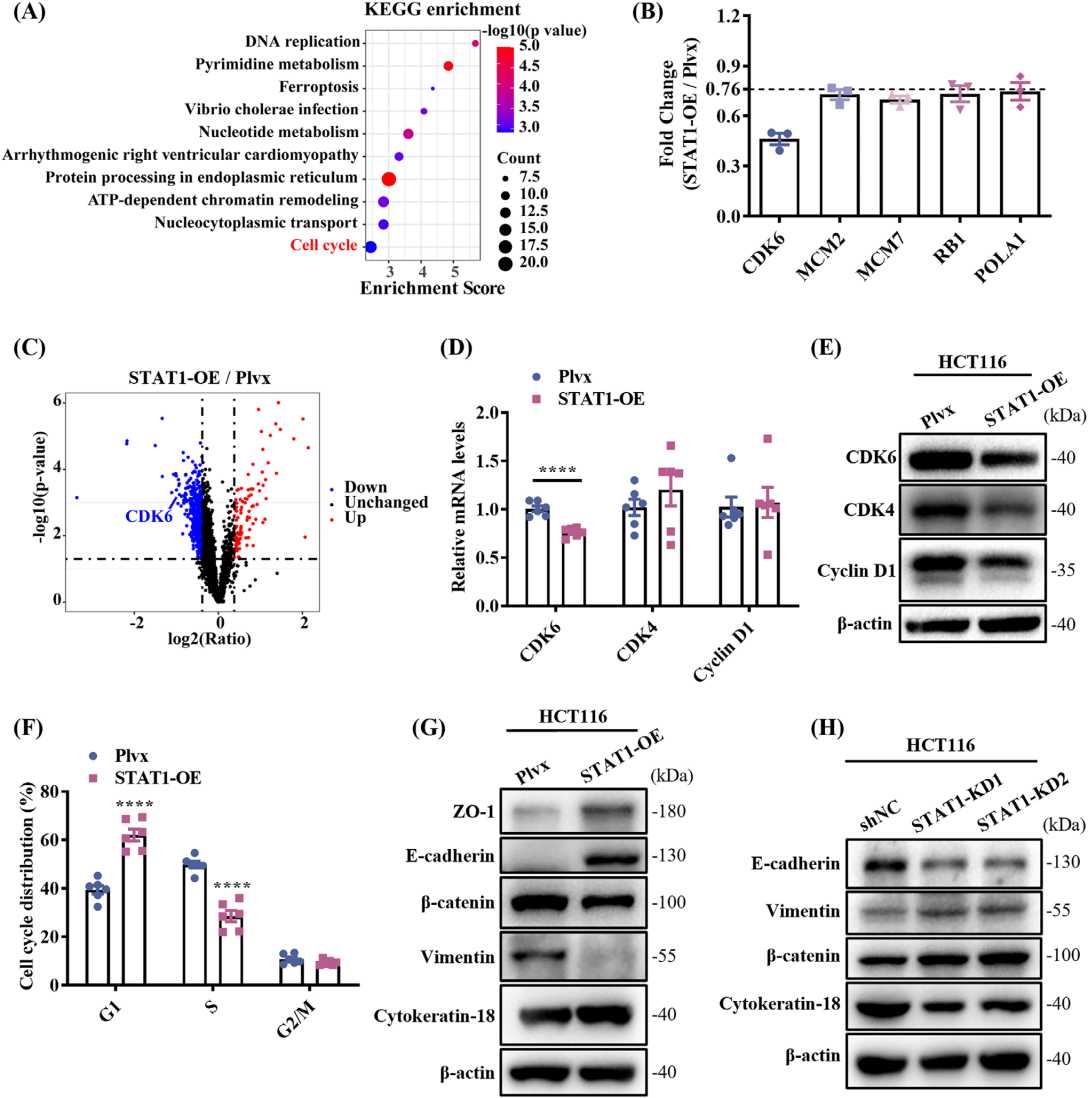
Signal transducer and activator of transcription 1 (STAT1) overexpression induces cell cycle arrest and inhibits epithelial–mesenchymal transition (EMT). (A) Kyoto Encyclopedia of Genes and Genomes (KEGG) pathway analysis of differentially expressed proteins (DEPs). (B) Five proteins related to cell cycle were reduced in STAT1‐overexpressing cells. (C) Volcano plot of the distribution of DEPs. CDK6 was significantly downregulated after STAT1 overexpression. (D) Quantitative polymerase chain reaction (qPCR) validation of CDK6, CDK4, and Cyclin D1. (E) Western blotting validation of CDK6, CDK4, and Cyclin D1. (F) STAT1 overexpression induces cell cycle arrest in the G1 phase. (G, H) Western blotting validation of EMT markers in STAT1‐overexpressing cells (G) or knockdown cells (H). Means ± SEM, ****p < 0.0001; Student's *t*‐test.

Next, proteomic changes upon STAT1 overexpression in HCT116 cells were analyzed to elucidate the function of STAT1 in CRC progression. A total of 6499 proteins were identified, of which 4855 proteins were quantified (FDR of protein <0.01, sum PEP score ≥5, unique peptide ≥2; Table S4). The experiment consisted of three biological replicates with high correlation (Figure S6A). Based on the distribution of coefficients of variation, proteins with a fold change greater than 1.3 or less than 0.76 were considered DEPs (Figure S6B), which included 97 upregulated proteins and 515 downregulated proteins. KEGG pathway analysis revealed that these proteins were significantly enriched in the cell cycle pathway (Figure 6A). Furthermore, the pathway map revealed that the expression levels of multiple proteins within the cell cycle pathway were significantly reduced, leading to the suppression of the pathway's activity (Figure S6D). Among the proteomic DEPs, a notable downregulation of several proteins linked to the cell cycle and DNA replication was observed in cells with reduced STAT1 expression, as depicted in Figure 6B. The volcano plot particularly highlighted a significant downregulation of CDK6 (Figure 6C), indicating its potential role in the observed phenotype. Expression levels of CDK6, CDK4, and Cyclin D1, which are critical mediators of the cellular transition to the S phase, were further verified by qPCR and WB (Figure 6D,E). Both mRNA and protein levels of CDK6 decreased, in agreement with the proteomic results. However, only the protein levels of CDK4 and Cyclin D1 were decreased, suggesting that CDK6 plays an important role in regulating the cell cycle in CRC. To determine the effect of STAT1 on the cell cycle, cells were stained with propidium iodide for flow cytometric analysis. The results show that more than 60% of STAT1 overexpression cells were maintained in the G1 phase, compared to less than 40% in the control group (Figure 6F), confirming that STAT1 inhibited cell proliferation through cell cycle arrest.

In addition, GO annotation revealed that the DEPs were enriched in pathways related to cell–cell adhesion and cadherin binding (Figure S6C). Tumor progression often results from a process called the epithelial–mesenchymal transition (EMT), in which cells with strong cell–cell adhesion are transformed into cells with motile and aggressive properties.^22^ By characterizing the expression of several EMT markers and cellular phenotypes, we confirmed that STAT1 overexpression inhibited EMT. The results showed that the expression levels of mesenchymal cell markers, such as β‐catenin and vimentin, were lower, whereas the expression levels of epithelial cell markers, such as E‐cadherin, ZO‐1, and cytokeratin 18, were higher in the HCT116‐STAT1‐OE cells compared with the control cells (Figure 6G). The expression of E‐cadherin and cytokeratin 18 was decreased, while the expression of β‐catenin and vimentin was upregulated in STAT1 knockdown cells (Figure 6H).

## 3 DISCUSSION

STAT1 functions as a tumor suppressor in various malignancies.^23^ During the initial stages of CAC, STAT1 deficiency leads to the accumulation of granulocytic cells induced by IL‐17, thereby exacerbating the inflammatory responses.^24^ Promoter CpG island (CpG) hypermethylation has been shown to be associated with transcriptional silencing of tumor suppressor genes in cancer cells^25^ and is particularly pronounced in CRC.^26^ STAT1 binds to TET methylcytosine dioxygenases 2 (TET2) and promotes chemokine and PD‐L1 gene hydroxymethylation, and reduced Ten‐eleven translocation (TET) activity allows tumors to evade antitumor immunity and resist anti‐PD‐L1 therapy.^27^ Therefore, when the expression of STAT1 is reduced, it may not bind TET2 efficiently, promoting CRC development. CDK4/6 forms complexes with Cyclin D1 to phosphorylate retinoblastoma protein and activate E2F transcription factors, thereby promoting cell entry into the S phase of the cell cycle.^28^ However, STAT1 overexpression reduced CDK6 expression at both the transcriptional and translational levels without affecting the mRNA expression of CDK4 and Cyclin D1 (Figure 6D,E). IFN‐γ stimulation could reduce the interaction of Cyclin D1 with STAT1, and induce its degradation via the proteasome‐dependent pathway,^29^ which may be the reason for the degradation of CDK4 and Cyclin D1 after STAT1 overexpression. A recent article reported that the knockdown of Cyclin D1 resulted in a decrease in STAT1 mRNA levels, but the study did not detect STAT1 protein levels,^30^ so how STAT1 regulates Cyclin D1 expression remains to be investigated further. Recently, it has been shown that IFN‐γ induces STAT1‐dependent upregulation of miR‐29, which reduces CDK6 expression and induces G1‐arrest in melanoma cells.^31^ This suggests that CDK6 is a target of STAT1.

Excessive activation of the Wnt/β‐catenin signaling pathway is one of the characteristics in CAC that promotes tumorigenesis and induces EMT.^32^ In this study, β‐catenin expression was downregulated and the expression of epithelial markers such as E‐cadherin were upregulated in HCT116‐STAT1‐OE cells (Figure 6G), indicating that STAT1 overexpression inhibited EMT. During EMT, dysfunction of cell–cell adhesion and an increase in cell motility promote tumor metastasis.^33^ Thus, the prevention of EMT appears to contribute to the antitumorigenic activity of STAT1.

Our previous studies demonstrated that cysteine trioxidation is a crucial modification that triggers protein degradation.^15^, ^34^ In this study, we found that H_2_O_2_ induces the oxidation of Cys155 in STAT1 to sulfonic acid, which contributes to its degradation (Figures 3A and 4A). Recently, new protein degradation pathways have been discovered, such as the midnolin‐mediated proteasome pathway,^35^ in which PLD6 promotes LDLR degradation in a phosphatidic acid‐dependent manner.^36^ This allowed us to consider whether the oxidative degradation of proteins is cell type‐specific or pervasive, which requires validation and detailed studies using more cell lines. Altering Cys155 to alanine halted ROS‐induced STAT1 degradation, suggesting that Cys155 plays a crucial role in maintaining STAT1 stability. The STAT proteins' coiled‐coil domain, spanning residues 136–317, is composed of four α‐helices and presents a predominantly hydrophilic surface for engaging with various crucial regulatory proteins.^37^ N‐myc interactor interacts with all STATs, except STAT2, via the coiled‐coil region, which enhances CBP/p300 recruitment, thus augmenting transcriptional activity.^38^ Therefore, oxidation of Cys155 in STAT1 likely impacts its protein interactions and intracellular stability, highlighting the role of cysteine trioxidation in regulating STAT1 stability.

NMN supplementation enhances the cellular antioxidant capacity and reduces inflammation.^39^ Recent studies indicate that NMN, when administered intraperitoneally, mitigates oxidative stress in mice's skin and liver by stimulating the AMPK pathway and preventing Ultraviolet radiation b (UVB)‐induced skin damage.^40^ Moreover, NMN inhibits hepatic stellate cell activation by preventing the oxidative degradation of 15‐PGDH, thereby preventing liver fibrosis.^41^ Our findings demonstrate that NMN supplementation not only reduced ROS and prevented STAT1 degradation in HCT116 cells, but also alleviated AOM/DSS‐induced oxidative damage, restored STAT1 levels in colon tissue, and lowered inflammatory markers. Studies have shown that NMN supplementation enhances NK cell cytotoxicity, promotes the clearance of dysfunctional cells, and boosts immune activity in mice.^42^ In addition, NMN treatment inhibits arginase‐1 and effectively prevents T cell dysfunction, thus preserving the anticancer function of T cells and preventing tumor occurrence in mice with normal immune function.^43^ Moreover, NMN can reduce Lipopolysaccharide (LPS)‐induced macrophage inflammation and proinflammatory cytokine secretion.^44^ Therefore, whether NMN also activates immune cells to combat tumors and prevent AOM/DSS‐induced CRC is worth exploring.

Our research has advanced the field, but it has limitations. In particular, the main degradation pathway of oxidatively damaged proteins in cells is through the 20S proteasome,^45^ and the Pa28(11S) regulatory factor enhances the selective degradation of oxidized proteins by the 20S proteasome.^46^ However, the mechanisms underlying the oxidative degradation of STAT1 are complex and not fully understood. Future research should take a systematic approach to uncover the intricate cellular responses to oxidative stress. Moreover, we found that Cys155 on STAT1 is oxidized to sulfonic acid, but no peroxidation was observed in mouse tissues, possibly owing to the low abundance of STAT1 as a transcription factor. In future studies, we plan to use data‐independent acquisition to scan all ions, identifying relatively low‐abundance proteins and modified peptides in complex samples. In our study, NMN was given preventively, but its potential to reverse inflammation or halt the “inflammation–cancer transition” remains unclear. Prior research showed that NMN can reduce COX‐2 expression in macrophages, mitigating LPS‐induced inflammation.^44^ Since high COX‐2 levels in colorectal tissues correlate with poor outcomes in CRC,^47^ and COX‐2 inhibitors have demonstrated promising results in clinical trials,^48^ NMN might prevent CRC by inhibiting COX‐2. Future studies will test the effects of NMN on established intestinal inflammation in mice or in combination with other treatments to assess its potential for managing inflammation.

In conclusion, we found that STAT1 expression was lower in the AOM/DSS‐induced CAC model and in H_2_O_2_‐treated cells. Mechanistically, we demonstrated that H_2_O_2_‐induced oxidation of Cys155 in STAT1 to sulfonic acid contributes to its degradation through the ubiquitin–proteasome pathway. STAT1 overexpression induces cell cycle arrest and suppresses EMT, thereby inhibiting the proliferation and migration of CRC cells. Notably, NMN supplementation partially inhibited STAT1 degradation by reducing intracellular ROS levels. Furthermore, NMN pretreatment restored STAT1 expression and suppressed the development of CAC in the AOM/DSS mouse model. In summary, this study highlights the role of the oxidative modification of STAT1 in CRC and provides experimental evidence for the development of NMN‐based CRC prevention strategies.

## 4 METHODS

### 4.1 AOM/DSS‐induced CAC in mice

All animal experiments adhered to the guidelines of the Tsinghua University Laboratory Animal Research Center and were approved by the Institutional Animal Care and Use Committee. Male C57BL/6J mice, around 12 months old and weighing 42 ± 6 g, were housed in a specific pathogen‐free environment with ad libitum food and water. The CAC model was induced with a modified AOM/DSS protocol: 12.5 mg/kg AOM injection on day 1, followed by cycles of 2.5% DSS in drinking water for 6 days and sterile water for 12 days, repeated twice. Mice were sacrificed 12 days after the last DSS treatment for tissue collection, and the colons were removed, rinsed, measured, and checked for tumors.

Mice were divided into three groups: normal control (C, *n* = 5), AOM/DSS treated (A, *n* = 8), and AOM/DSS treated with NMN (AN, *n* = 8). NMN, at 500 mg/kg via intraperitoneal injection every other day, started 1 day before AOM and continued throughout. Disease progression was monitored biweekly based on body weight and stool consistency. For cell proliferation, BrdU (50 mg/kg, intraperitoneally) was given 2 h before sacrifice.

### 4.2 Colon histology

Colons were prepared as “Swiss‐rolls,” fixed in 4% paraformaldehyde (Solarbio) at 4°C overnight, and embedded in paraffin. Sections (5 µm) were sequentially cut from the entire block. Following deparaffinization and rehydration, the sections were stained with hematoxylin and eosin for histological assessment.

### 4.3 Immunohistochemistry (IHC)

Paraffin sections were dewaxed, rehydrated, and subjected to antigen retrieval by microwave heating. The peroxidase activity was quenched with 3% H_2_O_2_ for 10 min at 25°C, followed by blocking with goat serum for 30 min at 37°C. Anti‐Ki67 (Abcam, ab16667) and anti‐BrdU (Servicebio, GB12051) antibodies were used at a dilution of 1:200 and incubated overnight at 4°C. Horseradish peroxidase‐conjugated anti‐rabbit and anti‐mouse immunoglobulin G (IgG) were used as secondary antibodies. Ki67 and BrdU were stained using the 3,5‐diaminobenzidine peroxidase substrate kit (Servicebio, G1212‐200T), and the sections were then counterstained with hematoxylin and mounted with neutral gum. The evaluation was based on established criteria, scoring the proportion and intensity of positive staining, and the final score was calculated as the product of the two scores.^49^

### 4.4 Quantitative proteomic analysis

Quantitative proteomics using Tandem Mass Tag (TMT) labeling was performed on six cell samples, with triplicates from both control and STAT1‐overexpressing cells, and seven tissue samples from four Group A and three Group AN mice. Proteins were extracted from colon tissues or cells using radioimmunoprecipitation assay (RIPA) lysis buffer (Solarbio) containing a 1% protease inhibitor cocktail (Bimake). After centrifugation at 12,000 × *g* for 20 min at 4°C, supernatants were collected and precipitated with four times the volume of precooled ice acetone for 4 h at −20°C. Then, the supernatant was discarded after centrifugation at 8000 × *g* for 10 min at 4°C. The precipitate was redissolved in 8 M urea (Solarbio), protein concentrations measured using a Bicinchoninic acid (BCA) assay kit (Beyotime), and disulfide bonds reduced with 5 mM dithiothreitol (Solarbio) for 1 h at 25°C, followed by alkylation with 12.5 mM iodoacetamide (Sigma‐Aldrich) in the dark for 30 min. Proteins were then digested with trypsin (Promega) at a 50:1 substrate‐to‐enzyme ratio at 37°C overnight, then desalted using SepPak tC18 Cartridges (Waters).

Peptides were labeled with TMT, mixed, desalted, and separated into 12 fractions by high‐pH reversed‐phase chromatography for liquid chromatography with tandem mass spectrometry (LC–MS/MS) analysis. The 120‐min gradient elution was performed at 0.30 µL/min using a high‐performance liquid chromatography (HPLC) system connected to an Orbitrap Exploris 480 Mass Spectrometer. The peptides were analyzed on a homemade C‐18 resin column with a mobile phase containing 0.1% formic acid and 80% acetonitrile. The mass spectrometer was operated in the data‐dependent mode with Xcalibur 2.1.2 software, recording a full‐scan mass spectrum (300–1800 *m*/*z*, 70,000 resolution) and 20 MS/MS scans at 27% normalized collision energy. Raw data were searched against UniProt human (20,308 sequences, downloaded 2022‐04‐18) or mouse (17,090 sequences, downloaded 2022‐01‐13) databases using the SequestHT engine with Proteome Discoverer 2.3 software. The search parameters included fixed modifications: carbamidomethylation on cysteine, TMT 6‐plex on lysine/N‐termini, variable oxidation on methionine, and two missed trypsin cleavages. Mass tolerances were set at 20 ppm for the precursors and 0.02 Da for fragments. Peptide FDR was calculated using Percolator, with Peptide‐spectrum matches (PSMs) with *q* < 1% considered correct. FDR for proteins was set at 0.01, based on searches against the decoy database, and unique peptides were assigned solely to specific protein groups.

### 4.5 Western blot

Tissues and cells were lysed and sonicated in RIPA buffer containing protease inhibitors. Supernatants were collected and quantified, and equal amounts of protein were electrophoresed on a 7.5% SDS‐PAGE gel and transferred to a 0.45 µm Polyvinylidene difluoride (PVDF) membrane. WB analysis was performed according to a conventional protocol. Finally, a ChemiDoc XRS + SYSTEM (Bio‐Rad) was used for imaging. STAT1, p‐STAT1 (Tyr701), p‐STAT3 (Ser727), vimentin, β‐catenin, flag‐tag, and β‐actin antibodies were sourced from Cell Signaling Technology (catalog numbers 9172S, 9167S, 9134S, 5741S, 8480S, 2368S, and 4970S). Antibodies against STAT3, ZO‐1, and CDK4 were purchased from Proteintech (80149‐1‐RR, 21773‐1‐AP, and 11026‐1‐AP, respectively). Cytokeratin 18 and ubiquitin antibodies were purchased from Millipore (MAB3236 and 05–944), and the E‐cadherin antibody was purchased from Abcam (2368S). Antibodies against CDK6 and Cyclin D1 were obtained from ZENBIO (R23891 and R380999, respectively).

### 4.6 Cell culture and construction of cell lines

HEK293, HCT116, Caco2, and A549 cells were purchased from the Cell Bank of the Chinese Academy of Sciences and maintained in our laboratory. HEK293 and Caco2 cells were cultured in Dulbecco's modified eagle medium (DMEM) (WISENT INC., 319‐005‐CL) with 10% fetal bovine serum (FBS; PAN‐Biotech, P30‐3302), 1% streptomycin‐penicillin (WISENT INC., 450‐201‐el) at 37°C in 5% CO_2_ humidified incubator. HCT116 and A549 cells were cultured in RPMI 1640 medium (WISENT Inc., 350‐600‐CL) supplemented with the same reagents.

HCT116 cells were transiently transfected with the DNA constructs using a liposomal reagent (YEASEN, 40802ES02). STAT1 knockdown plasmid (pLKO‐puro) was obtained from Tsinghua University's Center for Biomedical Analysis. The shRNA sequences used are listed in Table S5. The pLVX‐IRES‐ZsGreen1 vector was used for overexpression, and STAT1 mutant plasmids with C155A or C155D substitutions were generated using a Seamless Cloning Kit (Beyotime, D7010M). Cells and packaging components were transfected into HEK293 cells with PEI. After 6 h, the medium was changed, and the supernatant was collected after 48 h. It was concentrated with PEG6000 and used to infect HCT116 cells with polybrene for 12 h. Overexpression was detected by flow cytometry, and knockdown was selected with 2 µg/mL puromycin, confirmed by WB.

### 4.7 qPCR

Total RNA was extracted using TRIzol (TIANGEN), and 2 µg of each sample was converted into cDNA using a reverse transcription kit (CWBIO). qPCR was conducted using SYBR green on a Roche LightCycler 96 System, with β‐actin (ACTB) as the internal control. The 2^(−ΔΔCt)^ method was employed to calculate gene expression, and primer sequences are detailed in Table S6.

### 4.8 Cell proliferation assay

The cells were plated in 96‐well plates and cultured as required. Cell proliferation was measured using the CCK‐8 assay (ApexBio Technology). After media removal and washing with phosphate buffered saline (PBS), cells were incubated with 10% CCK‐8 in media at 37°C for 1.5 h. The cell number was determined by measuring the absorbance at 450 nm.

### 4.9 Colony formation assay

Cells (1 × 10^3^) were plated in triplicate in six‐well plates and cultured for 7 days. Colonies were washed with PBS, fixed with paraformaldehyde, and stained with 0.1% crystal violet for analysis.

### 4.10 Wound‐healing assay

Cells were seeded at a density of 2 × 10^5^ cells/well in a 24‐well plate with predrawn horizontal lines. Once confluent, a straight scratch was made through the monolayer, followed by three washes with PBS to remove the nonadherent cells, revealing a clear gap. The scratch widths were measured and photographed under a microscope. After replacing the medium, the cells were incubated and the scratch width was remeasured after 24 h.

### 4.11 Transwell assay

Transwell assays were performed using Corning chambers. For migration, 5 × 10^4^ cells in 200 µL 5% FBS medium were added to the upper chamber, with 700 µL 20% FBS in the lower chamber. After 24 h, cells were fixed with 4% formaldehyde, stained with 0.1% crystal violet, and washed with PBS. The cell counts were determined microscopically. For invasion assays, the chambers were precoated with 1 mg/mL Matrigel before cell seeding.

### 4.12 Cell cycle determination

STAT1 overexpression and control cells were collected, washed in PBS, and fixed in 70% cold ethanol overnight at −20°C. After centrifugation at 800 rpm for 5 min, the ethanol was removed. Cells were resuspended in PBS, treated with 50 µL of 100 µg/mL RNaseA (TIANGEN), then stained with 40 µg/mL propidium iodide (Leagene) at 37°C for 30 min. Cell cycle analysis was performed using a BD LSR Fortessa SORP flow cytometer (BD Biosciences).

### 4.13 Detection of cellular ROS

ROS levels in the control and NMN‐treated HCT116 cells were measured using a Dihydroethidium (DHE) fluorescent probe (KeyGEN Biotech) following the manufacturer's instructions. Cells were harvested, washed with PBS, and incubated with 20 µM DHE at 37°C for 30 min. The fluorescence of 20,000 cells was assessed using a flow cytometer, with ROS levels indicated by the mean fluorescence intensity at 640 nm.

### 4.14 Immunoprecipitation followed by LC–MS/MS analysis

As described in Section 4.4, H_2_O_2_‐treated and untreated STAT1‐OE HCT116 cells were harvested. The protein supernatant was incubated with Anti‐FLAG Affinity Gel (Bimake) overnight at 4°C, followed by four washes with lysis buffer and boiling with 2 × loading buffer (CWBIO). Equal sample volumes were subjected to SDS‐PAGE and stained with Coomassie Blue. The target bands were excised for in‐gel digestion, alkylated with iodoacetamide, and digested with trypsin. Peptides were analyzed by LC–MS/MS using a Q Exactive HF mass spectrometer. The parameters mirrored TMT‐based proteomics, and data were searched against the UniProt human STAT1 sequence using SequestHT via Proteome Discoverer 2.1. The FDR was set to 0.01, with search parameters including variable modifications of cysteine (trioxidation +47.985 Da, dioxidation +31.990 Da) and oxidation of methionine and cysteine (+15.995 Da).

### 4.15 Determination of 8‐hydroxy‐2′‐deoxyguanosine

We measured 8‐OHdG levels using the GERSION ELISA kit. Tissues were lysed, centrifuged at 3000 rpm for 10 min, and 10 µL of supernatant mixed with 40 µL diluent was added to a 96‐well plate alongside standards. Horseradish peroxidase‐labeled antibody was added, followed by incubation at 37°C for 1 h and washing. Substrate A and B were added, and after 15 min in the dark, termination solution was added. The absorbance at 450 nm was measured within 15 min to determine 8‐OHdG concentrations using a standard curve.

### 4.16 Statistic method

GraphPad Prism 9.0 and R version 4.2 were utilized for statistical analysis and data visualization. GO and KEGG enrichment were conducted with the R package clusterProfiler. Protein–protein interaction analysis was done using the STRING database (https://string‐db.org/), with a medium confidence level (0.4), considering both functional and physical associations, and visualized with Cytoscape 3.10.0. *p* values were adjusted using the Benjamini–Hochberg method for multiple testing, with significance set at *p* < 0.05. Data are shown as mean ± SEM. Protein abundance ratios were compared using *p* values derived from biological replicates. Variance equality was assessed by the *F*‐test prior to the Student's *t*‐test, and one‐way analysis of variance (ANOVA) was applied for multiple group comparisons.

## AUTHOR CONTRIBUTIONS

Research design: Ting Li and Haiteng Deng. Experimental operations: Ting Li, Chengting Luo, Zongyuan Liu, and Jinyu Li. Animal experiments thanks to the guidance of Chengting Luo. Mass spectrometry data availability: Meng Han and Yuling Chen. Results analysis and visualization: Ting Li and Haiteng Deng. Writing: Ting Li, Ran Zhang, and Haiteng Deng. All the authors read and approved the final manuscript.

## CONFLICT OF INTEREST STATEMENT

The authors declare no conflicts of interest.

## ETHICS STATEMENT

Animal experiments in this study were conducted in accordance with the welfare ethics review approval document (2022) No. 334 of the Laboratory Animal Use and Management Committee of the Laboratory Animal Center of Tsinghua University. Transcriptomic data for STAT1 in colorectal cancer patients were obtained from the TCGA database (https://portal.gdc.cancer.gov/).

## Supporting information



Supporting Information

Supporting Information

## Data Availability

All the raw data of mass spectrometry are available in iProx website (https://www.iprox.org), in which the proteomics data discussed in this publication are accessible through IPX0006321000.
