# Supplementary material for: Nicotinamide mononucleotide protects STAT1 from oxidative stress‐induced degradation to prevent colorectal tumorigenesis
Source: MedComm (2020). 2024 Nov 21;5(12):e70006. doi: 10.1002/mco2.70006 (PMC11581775; doi:10.1002/mco2.70006)
Supplement: Supplementary file 1 — Supporting Information [file MCO2-5-e70006-s002.docx]

**Nicotinamide mononucleotide protects STAT1 from oxidative stress induced degradation to prevent** **colorectal tumorigenesis**

Ting Li ^a, b, #^, Chengting Luo ^a, c, #^, Zongyuan Liu ^a^, Jinyu Li ^a^, Meng Han ^a^, Ran Zhang ^a^, Yuling Chen ^a^, Haiteng Deng ^a,*^

^a^ MOE Key Laboratory of Bioinformatics, Center for Synthetic and Systematic Biology, School of Life Sciences, Tsinghua University, Beijing, 10084, China

^b^ School of life science and technology, Wuhan Polytechnic University, Wuhan, 430048, China

^c^ School of Life Science, Yunnan University, Yunnan, 650091, China

**^*^To whom correspondence should be addressed:**

Haiteng Deng, Prof

Tel: 8610-62790498

E_mail: dht@mail.tsinghua.edu.cn

**^#^These authors contribute equally to this work.**


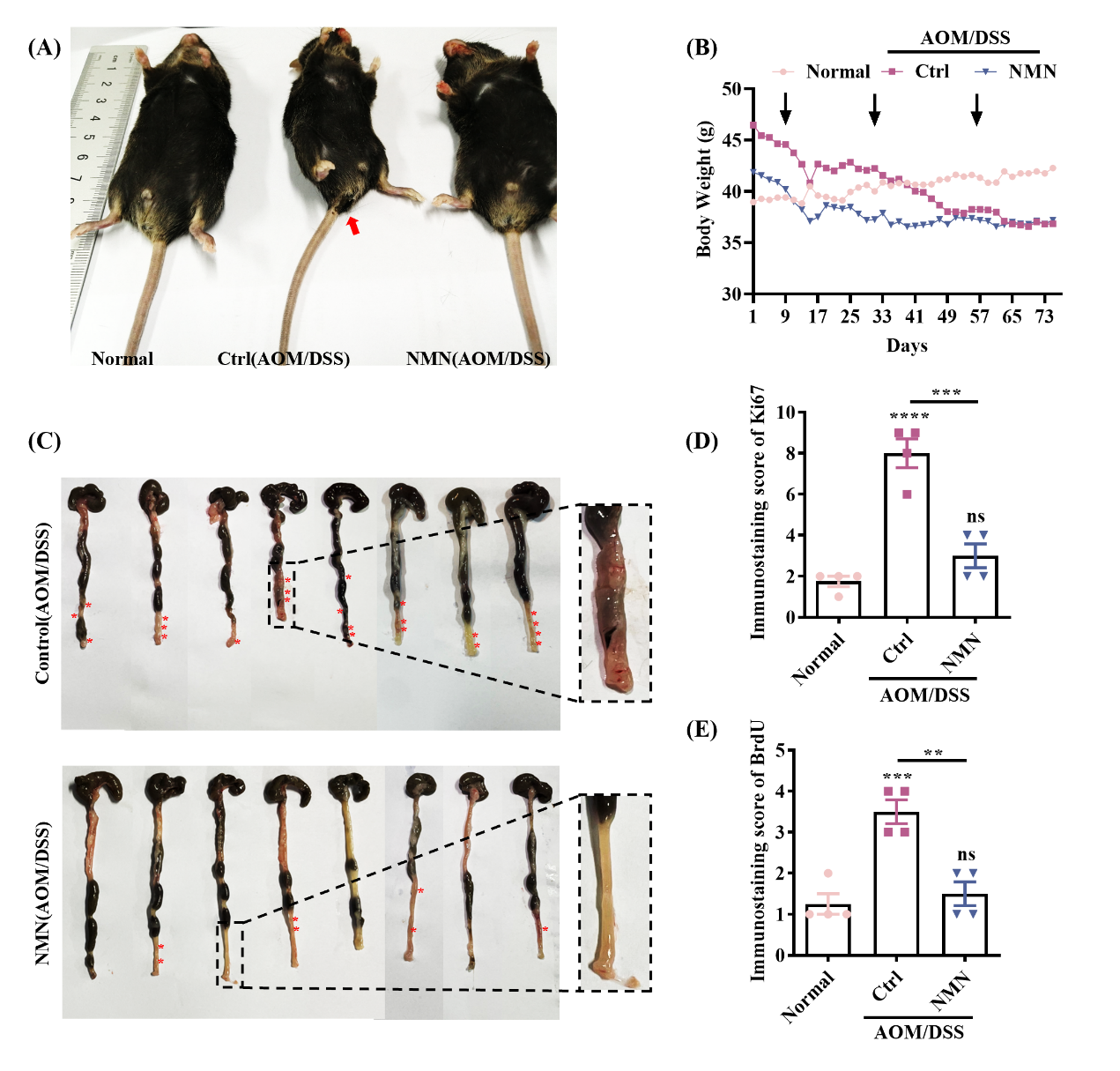


**Supplementary Figure 1. NMN supplementation reduces the colon tumorigenesis. Related to Figure 1.**

(A) AOM/DSS treatment induced diarrhea in mice, whereas no diarrhea was observed in mice from the normal group and the AOM/DSS NMN-treated group. (B) Mouse body weight (shown as the mean value) was monitored throughout the experiment. 2.5% DSS treatment caused short-term weight loss in mice. Black arrows indicate the start time points of DSS treatment cycles. (C) Colorectal tissues of mice treated with AOM/DSS. The enlarged part showed that the colorectal tissue damage in the control group was more serious, while the colorectal tissue in the NMN treatment group was more intact. The location of the tumor nodules were marked with red asterisks. The histological scores of Ki67 (D) and BrdU (E). Means ± SEM, **P < 0.01, ***P < 0.001, ****P < 0.0001; ns, not significant; one-way ANOVA test.


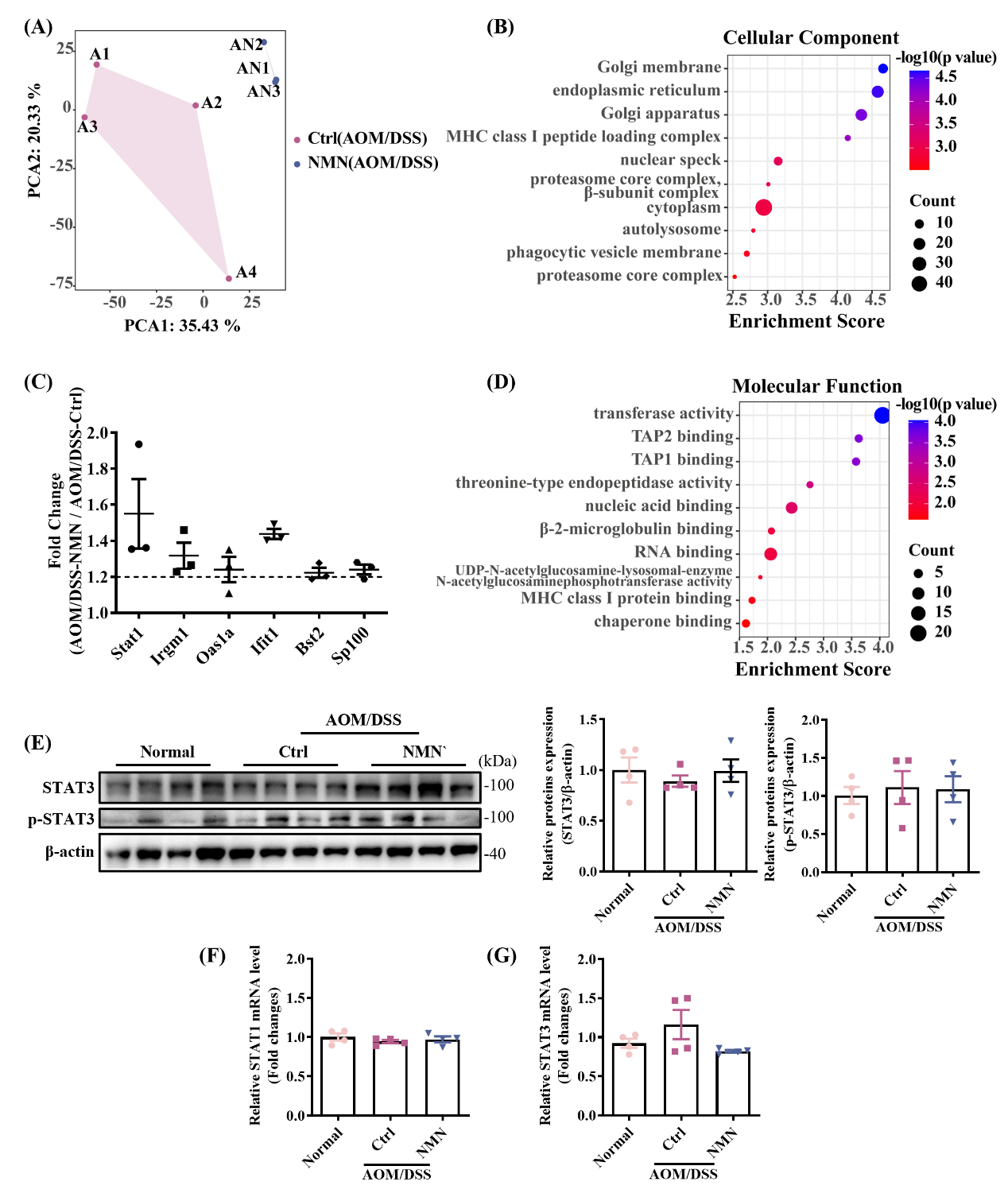


**Supplementary Figure 2. The proteomic results of mice colorectal tissues. Related to Figure 2.**

(A) PCA of protein expression in the control and NMN-treated groups under AOM/DSS condition based on the abundance of 4,706 proteins. (B) Six proteins responded to IFN stimulation were increased by NMN supplement. (C, D) GO annotation of DEPs. (E) Western blotting verification of STAT3 and phosphorylated STAT3. STAT1 (F) and STAT3 (G) mRNA expressions in three groups.

**
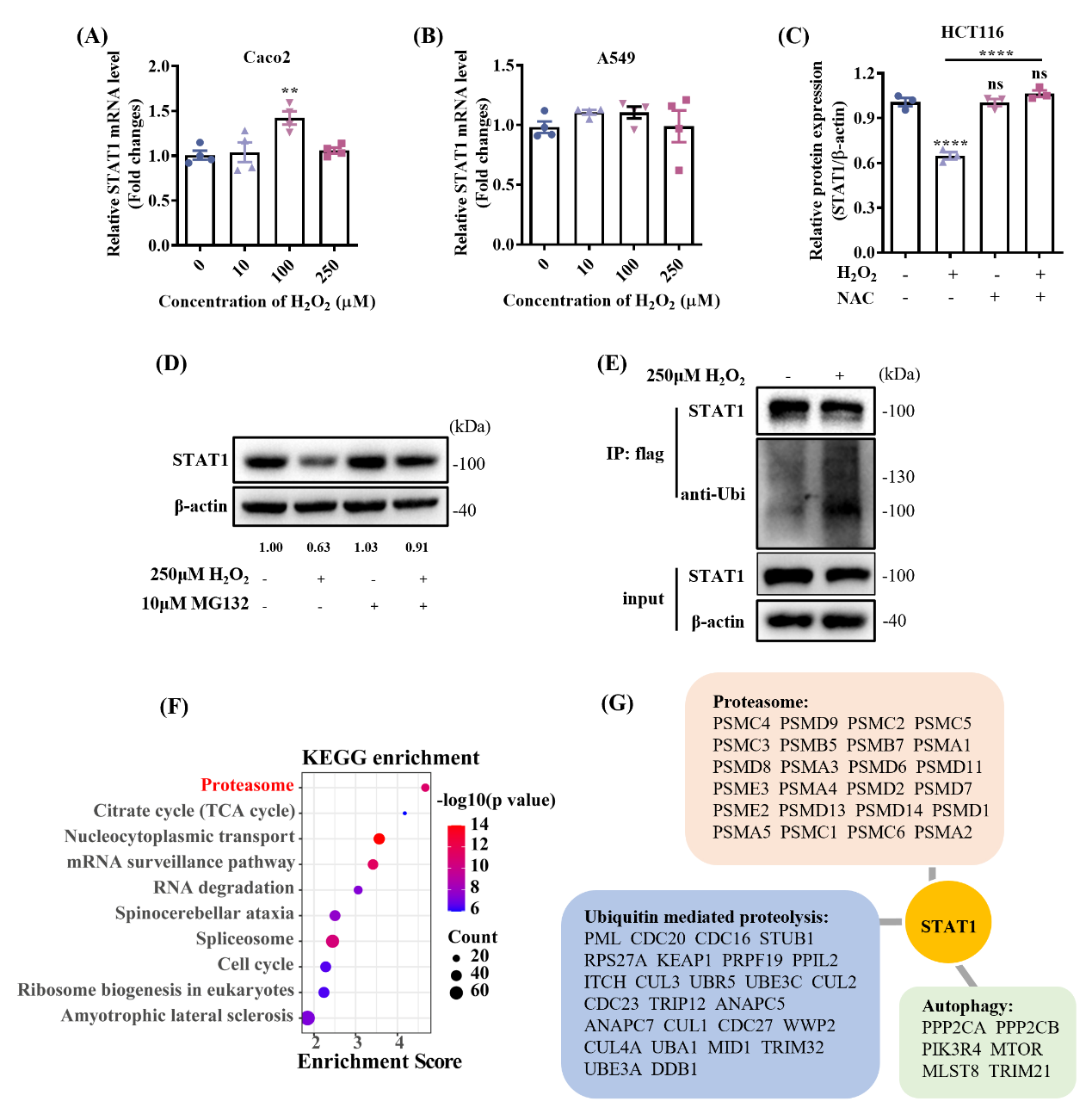
**

**Supplementary Figure 3. Proteasome pathway involved in ROS-induced STAT1 degradation. Related to Figure 3.**

qPCR results showed that the STAT1 mRNA levels did not change under H_2_O_2_ treatment in Caco2 (A) and A549 (B) cells. (C) NAC inhibits H_2_O_2_-induced reduction of STAT1. (D) The expression of STAT1 in untreated and H2O2-treated HCT116 cells with or without pretreatment with 10 μM MG132. (E) H_2_O_2_ treatment decreased the protein expression level of STAT1 and increased the ubiquitination level of STAT1 in HCT116-STAT1-OE cells. (F) KEGG pathway analysis of DEPs. (G) Binding partners of STAT1 under H2O2 treatment in HCT116-STAT1-OE cells. Means ± SEM, **P < 0.01, ****P < 0.0001; ns, not significant; one-way ANOVA test.


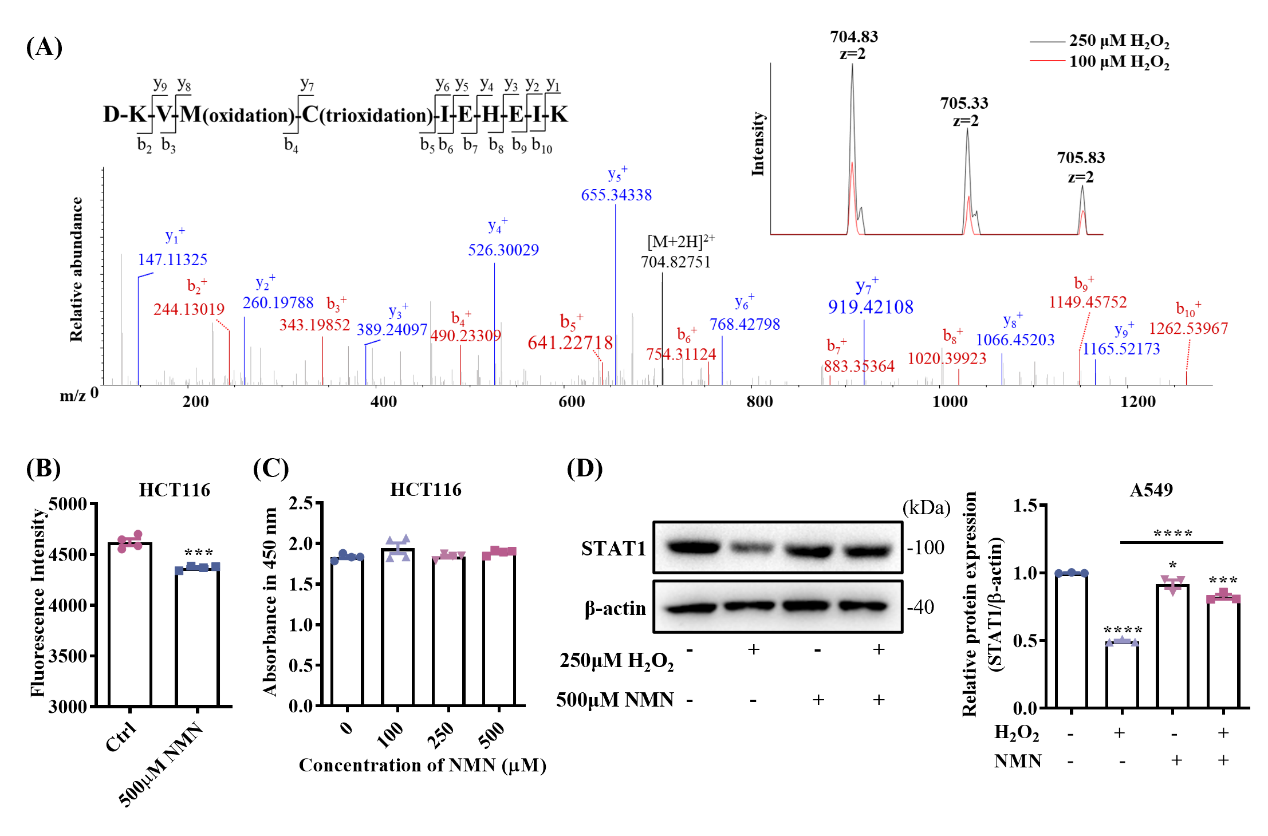


**Supplementary Figure 4. NMN supplementation inhibits the oxidative degradation of STAT1. Related to Figure 4.**

(A) Tandem mass spectrometry spectrum of the sulfonated peptide showing the identification of Cys155 modifications in STAT1 in the presence of different concentrations of H_2_O_2_, with inset showing ion intensity of the peptide. (B) NMN treatment induced lower ROS production in HCT116 cells as measured using a DHE fluorescent probe. (C) The proliferation of HCT116 cells was not affected by NMN treatment for 12 hours. (D) 500 μM NMN treatment prevented the reduction of STAT1 protein induced by H_2_O_2_ treatment in A549 cells. Means ± SEM，*P < 0.05, ***P < 0.001, ****P < 0.0001; Student’s t-test or one-way ANOVA test.


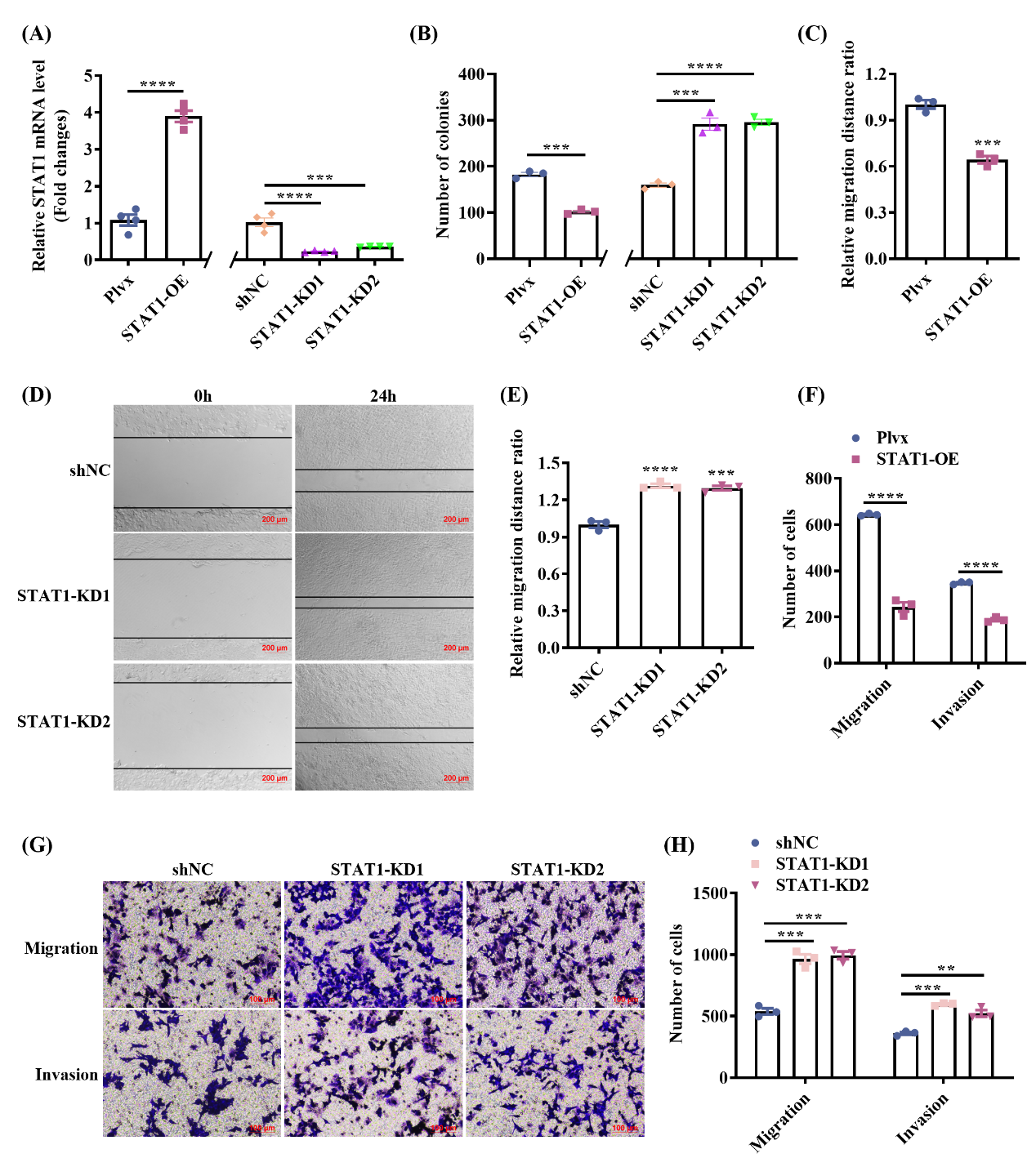


**Supplementary Figure 5. STAT1 overexpression induces cell cycle arrest.** **Related to Figure 5.**

(A) The verification of STAT1 overexpression or knockdown by qPCR. (B) Statistical analysis of colonies formation numbers of STAT1-OE or STAT1-KD cells. (C) Migration distance analysis of STAT1 overexpressed cells. (D) STAT1 knockdown promotes CRC cells migration. (E) Statistical analysis of migration distance of STAT1 knockdown cells. (F) Statistics of migrating and invading cells in STAT1 overexpressing cells. STAT1 knockdown cells were evaluated for migration and invasion using Transwell assays (G), with images at 100 µm scale and subsequent statistical analysis (H).


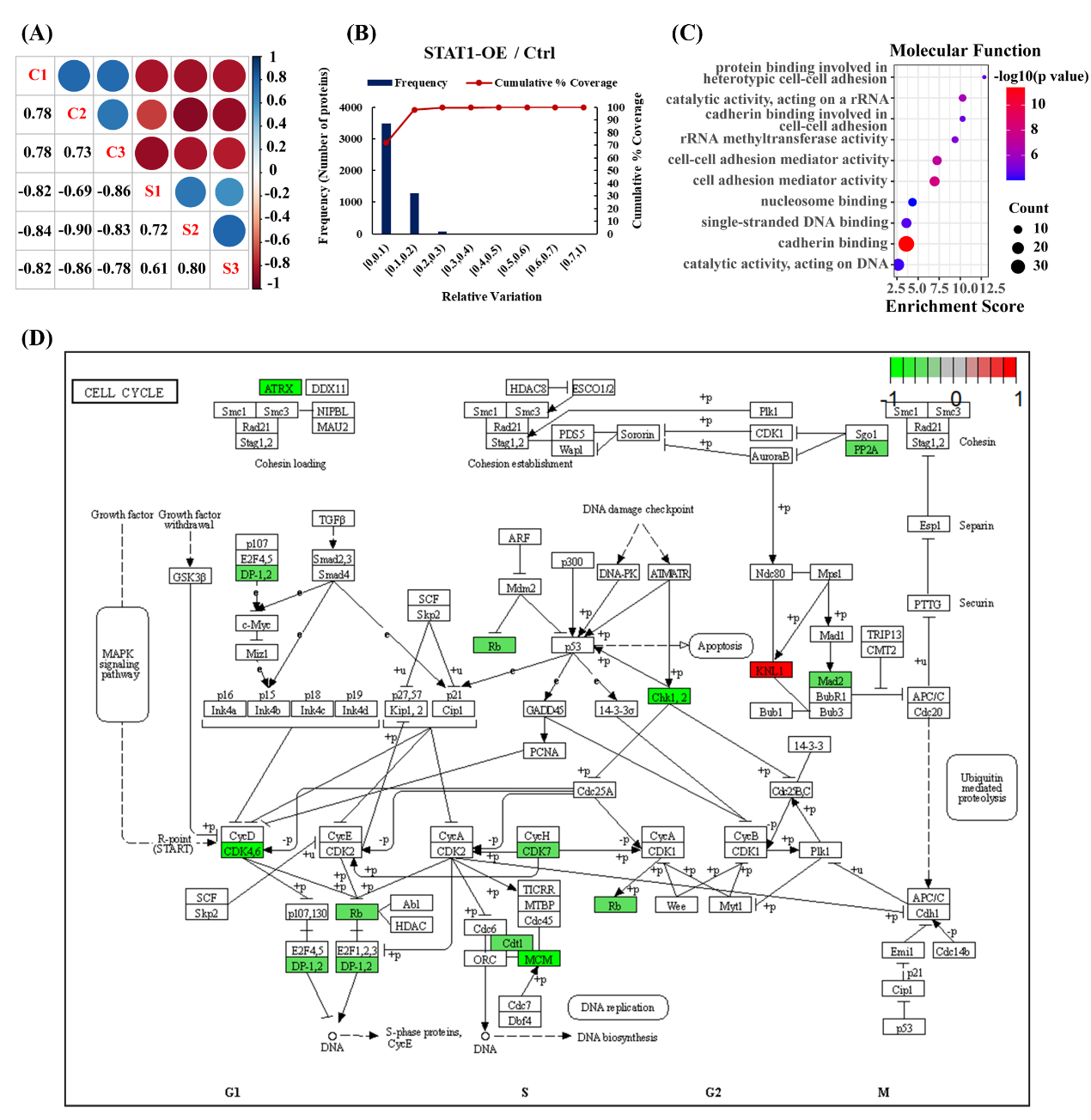


**Supplementary Figure 6. Proteomic results of STAT1 overexpression cells. Related to Figure 6.**

(A) The correlation matrix of all proteins in three biological replicates. The reproducibility was relatively high with the Pearson correlation coefficient larger than 0.6. C represents the HCT116-Plvx groups and S represents the HCT116-STAT1-OE groups. (B) Experimental variations of proteomics analysis between the control cells and the STAT1-OE cells. (C) GO annotation of DEPs. (D) Cell cycle pathway mapped with protein expression information using the clusterProfiler package by R. The red or green bar indicates up- or downregulation of proteins. Proteins in grey were not identified in this study.
